# 

**Published:** 1843-04-01

**Authors:** 


### CONTENTS.

A Course of Clinical Lectures, delivered at the Hôtel-Dieu, Paris, for the Session 1842–'43. By A. F. Chomel. Lecture 4.

Case 1. Puerperal metro-peritonitis three days after delivery; complicated with severe pectoral disease. Death —Autopsy, . . . . . . 61

Case 2. Organic affection of the heart—Hypertrophy of the ventricle, with valvular deficiency—General bronchitis—Abdominal tumour caused by the liver—Ascites, and infiltration of the lower extremities, and of the abdominal parietes, following protracted instrumental labour, . . . . 62

Sulphate of alumina as an antiseptic and detergent. By M.J.Pennypacker, M. D. . . . . . . . 63

*Jefferson Medical College.*

Clinic of Professor Mütter concluded. False Ankylosis of the elbow-joint, &c. 64

*Bibliographical Notices.*

An Essay on the use of Nitric Acid, as an Escharotic, in Certain Forms of Hemorrhoidal Affections; illustrated by Cases. By John Houston, M. D., M.R.I.A., &c. &c. . . . . . 66

A Treatise on the Structure, Economy, and Diseases of the Ear; being the Essay for which the Fothergillian gold medal was awarded by the Medico-Society of London. By George Pilcher, Late Lecturer on Anatomy, &c. &c. . . . . . . 68

Mortality of the Plague, . . . 69

*Editorial.*

The Medical Profession, . . . . 69

Invariable benefit of Mineral Acids in Dropsy, . . . . . . 70

Herpetic Pleuritis, . . . . . 70

*Retrospect of the Medical Sciences.*

Treatment of Vesico-Vaginal Fistula, . 71

Capacity of the Lungs, . . . . 71

Peculiar appearances in Cataract, . 72

Relative value of Quinine in large doses, as a remedy in Typhus, . . . . 72

